# Down-regulation of NTCP expression by cyclin D1 in hepatitis B virus-related hepatocellular carcinoma has clinical significance

**DOI:** 10.18632/oncotarget.10241

**Published:** 2016-06-23

**Authors:** Jingting Kang, Jie Wang, Jin Cheng, Zhiliang Cao, Ran Chen, Huiyu Li, Shuang Liu, Xiangmei Chen, Jianhua Sui, Fengmin Lu

**Affiliations:** ^1^ Department of Microbiology and Infectious Disease Center, School of Basic Medical Science, Peking University Health Science Center, Beijing 100191, P. R. China; ^2^ National Institute of Biological Sciences, Zhongguancun Life Science Park, Changping, Beijing, 102206, China; ^3^ Beijing Artificial Liver Treatment and Training Center, Beijing Youan Hospital, Capital Medical University, Beijing, 100069, P.R. China

**Keywords:** NTCP, hepatocellular carcinoma, survival, cyclin D1, HBV cccDNA

## Abstract

The sodium-dependent taurocholate cotransporter polypeptide (NTCP) has been identified as a liver specific functional receptor for the hepatitis B virus (HBV). Previous studies indicated that the expression of NTCP may be associated with the proliferation status of hepatocytes. However, the involvement of NTCP in hepatocellular carcinoma (HCC) cells proliferation remains unclear. In this study, we confirmed that NTCP was down-regulated in HCC tumor tissues compared with that in the adjacent non-tumor tissues (*P* < 0.0001). Clinically, lower expression of NTCP was correlated with poor post-surgery survival rate (*P* = 0.0009) and larger tumor tissue mass (*P* = 0.003) of HCC patients. This was supported by the finding that ectopic expression of NTCP in both HepG2 and Huh-7 cells could significantly suppress hepatocytes growth by arresting cells in G0/G1 phase. We also discovered that cyclin D1 could transcriptionally suppress NTCP expression by inhibiting the activity of NTCP promoter, while arresting HCC cells in G0/G1 phase by serum starvation could upregulate NTCP mRNA levels. This is the first study to report that the transcriptional inhibition of NTCP expression during cell cycle progression was mediated by cyclin D1. The down-regulated NTCP expression was associated with poor prognosis and lower HBV cccDNA level in HCC patients. Therefore, NTCP expression levels might serve as a novel prognostic predictive marker for post-surgery survival rate of HCC patients.

## INTRODUCTION

Hepatocellular carcinoma (HCC) is the sixth most common cancer and the second leading cause of cancer-related deaths worldwide [1]. Epidemiological evidence has clearly shown that chronic hepatitis B virus (HBV) infection is the major risk factor for the development of HCC [2]. It is known that chronic HBV infection and persistent inflammation may result in liver regeneration and cirrhosis, thereby inducing malignant transformation of liver cells [3, 4]. During the initiation and progression of hepatocarcinogenesis, multiple genetic and epigenetic events accumulated, leading to deregulated expression of various cellular genes [5, 6]. Therefore, identifying new molecular carcinogenic events in HCC will give guidance for the development of novel clinical treatment.

The Na^+^/taurocholate cotransporter polypeptide (NTCP, SLC10A1) is the main hepatocellular sodium-dependent uptake system for conjugated bile acids in human and rodent livers [7, 8]. It was originally discovered and cloned by researchers based on its ability to bind to and transfer bile acids from the blood to liver parenchymal cells. Recently, NTCP has been identified as a receptor of hepatitis B virus (HBV) by interacting with the N-terminus of the HBV large envelope protein [9]. NTCP is mainly or exclusively expressed in the liver, and this membrane protein is at least one of the factors determining the species specificity and hepatotropism of HBV [9, 10]. Expression of NTCP is maintained at a steady level in adult hepatocytes and dysregulation of its expression have been directly linked to various liver diseases, including inflammatory cholestasis, biliary cirrhosis and nonalcoholic steatohepatitis [11–13]. It has been reported recently that NTCP expression was significantly decreased in rat liver during the liver regeneration following a 90% hepatectomy, while the expression level of NTCP returned to normal immediately after liver organ reconstruction and hepatocytes entered into quiescence (G0 phase) from rapid cell proliferation [14]. This work revealed an association between NTCP transcription regulation and the proliferation status of hepatocytes, suggesting a mechanism whereby quiescent hepatocytes switching to rapid cell proliferation could silence NTCP expression.

One of the characteristics of cancer cells is uncontrolled proliferation and growth. Given the association between NTCP expression and cell proliferation status, it is reasonable to assume that the expression of NTCP might be suppressed in HCC tumor tissues. Indeed, recent works have identified the down-regulation of NTCP expression in HCC tumor tissues [13, 15]. However, the relationship between NTCP down-regulation and the development of HBV-related HCC are not fully understood. Here we reported that the decreased expression of NTCP in HCC tissues was significantly correlated with a poor post-surgery survival rate in HCC patients. Ectopic expression of NTCP could suppress cell growth in both HepG2 and Huh-7 cells by arresting cells in G0/G1 phase. Mechanistically, cyclin D1 could transcriptionally down-regulate NTCP expression, which might attribute to the decreased expression of NTCP in hepatocytes with rapid cell cycle progression including HCC tumor cells.

## RESULTS

### NTCP is down-regulated in HCC tumor tissues and correlated with the post-surgery survival rate of HCC patients

**Figure 1:**
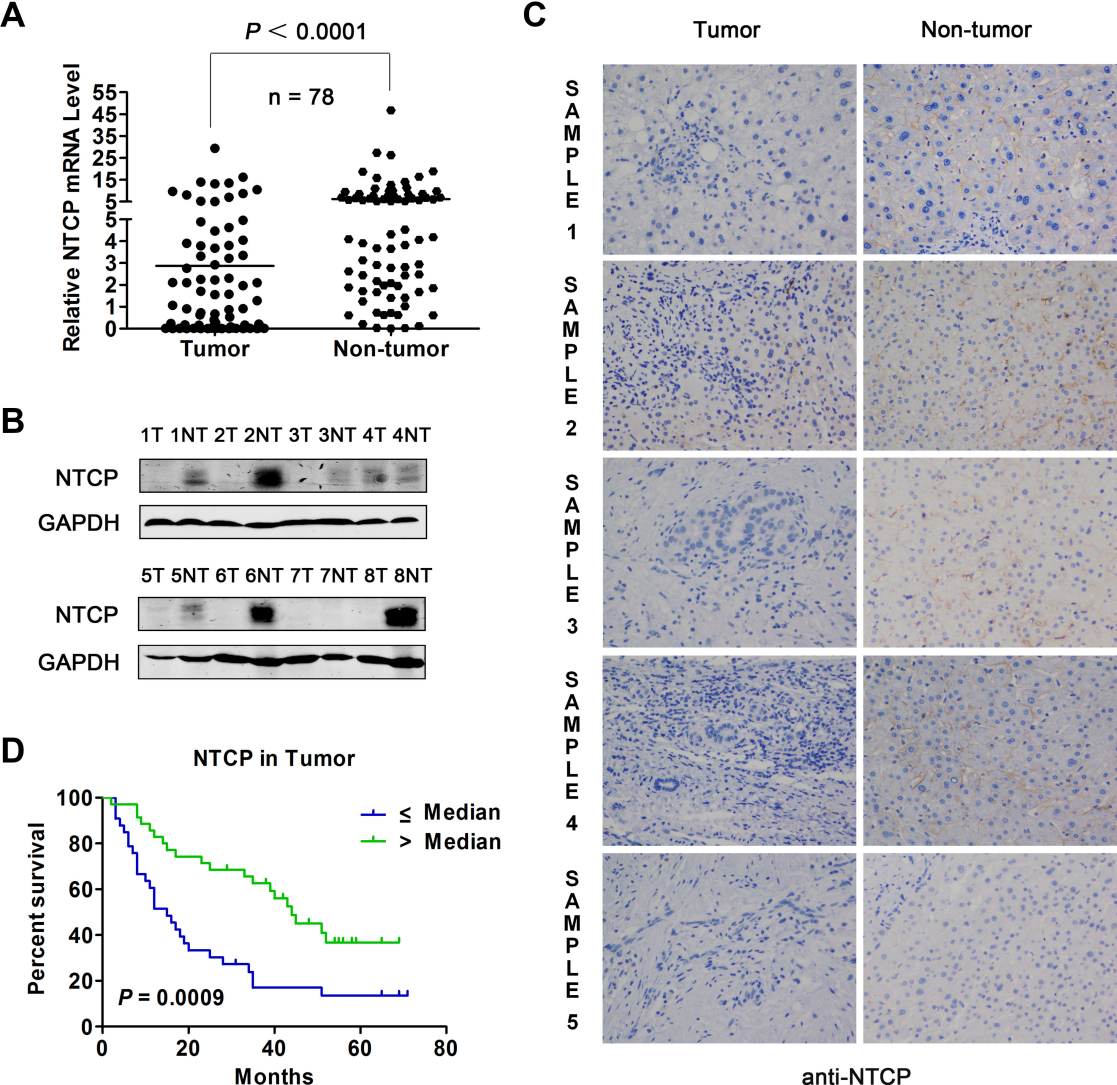
Expression of NTCP in HCC tumor tissues and its prognostic value for patient survival (**A**) The level of NTCP mRNA in 78 pairs of HCC tumor tissues and adjacent non-tumor tissues was assayed by real-time RT-PCR. (**B**) The protein levels of NTCP in 8 pairs of HCC liver tissues was assayed by Western blot using anti-NTCP monoclonal antibody P17-39. T: tumorous tissue; NT: adjacent non-tumor tissue. (**C**) The IHC study of NTCP was conducted in five pairs of HCC tumor tissues and adjacent non-tumor tissues (200×) using anti-NTCP monoclonal antibody P17-39. Immunostaining for NTCP reveals a positive membranes pattern in the four adjacent non-tumor tissues, whereas less/non-expressions in the five tumor tissues. (**D**) Survival rates of HCC patients with different levels of NTCP in tumor tissues were analyzed by Kaplan-Meier curve analysis. The total number of analyzed patients is only 69 of the study cohort of 78, due to the lack of follow-up data in some patients.

To explore the expression level of NTCP in HCC tissues, mRNA levels of NTCP in 78 pairs of HBV-related HCC tumor tissues and adjacent non-tumor tissues were tested using real-time RT-PCR assays. As shown in Figure 1A, NTCP expression was significantly down-regulated in HCC tumor tissues compared with that in adjacent non-tumor tissues (*P* < 0.0001). To detect the protein expression levels of NTCP in HCC tumor tissues and the adjacent non-tumor tissues, we generated a mouse monoclonal antibody (mAb), P17-39, against NTCP by hybridoma technology. As shown in the Supplementary Figure S1, P17-39 specifically recognized the human NTCP by Western blot (Supplementary Figure S1A), FACS (Supplementary Figure S1B) and immunofluorescent staining (Supplementary Figure S1C). Using the P17-39 mAb, we examined the protein level of the NTCP in HCC tumor tissues. The result showed that HCC tissues had markedly lower NTCP expression than that in the adjacent non-tumor tissues as evidenced by both Western blot (Figure 1B) and immunohistological (IHC) study (Figure 1C).

Next, the association of NTCP expression with patients’ post-surgery survival was analyzed in 69 patients for whom post-surgery follow-up information was available. Based on the median of NTCP mRNA levels in tumor tissues, patients were divided into NTCP high and low expression groups. Relative survival rate analysis using Kaplan-Meier curve revealed that patients with lower NTCP expression had a significantly poor overall survival rate than those with higher NTCP-expression (15.0 months vs. 44.0 months, *P* = 0.0009, Figure 1D). We also explored the correlation between NTCP expression and the clinicopathological features in these HCC patients. We found that patients with lower expression of NTCP exhibited a much larger tumor tissue mass (*P* = 0.003), implying a significantly reverse correlation between NTCP expression and HCC tumor size (Table 1). No significant difference of other clinicopathological features was observed between HCC patients with NTCP expression, including the Barcelona Clinic Liver Cancer (BCLC) stage, intrahepatic metastasis, serum αfetoprotein (AFP) level and tumor encapsulation. Taken together, these results suggested that the down-regulation of NTCP might be a novel significant prognostic factor for HCC patients.

**Table 1:** The correlation between the level of NTCP expression in HCC tissues and patients clinicopathological features

Clinicopathological features		Downregulation Case No. (%) *N* = 37	Non-downregulation Case No. (%) *N* = 38	*P* value
Gender	Male	30 (81.1%)	35 (92.1%)	0.191
	Female	7 (18.9%)	3 (7.9%)	
Age	≥ 50	22 (59.5%)	22 (57.9%)	1
	< 50	15 (40.5%)	16 (42.1%)	
Cirrhosis	Yes	31 (83.8%)	36 (94.7%)	0.153
	No	6 (16.2%)	2 (5.3%)	
	N/A	0	1 (2.6%)	
BCLC	A	16 (43.2%)	23 (60.5%)	0.162
	B + C	21 (56.8%)	14 (36.8%)	
	N/A	0	1 (2.6%)	
Portal vein invasion	Present	5 (13.5%)	5 (13.2%)	1
	Absent	32 (86.5%)	32 (84.2%)	
	N/A	0	1 (2.6%)	
Tumor size	≥ 5 cm	31 (83.8%)	20 (52.6%)	0.003
	< 5 cm	5 (13.5%)	18 (47.4%)	
	N/A	1 (2.7%)	0	
Tumor number	Single	25 (67.6%)	29 (76.3%)	0.449
	Multiple	12 (32.4%)	9 (23.7%)	
	N/A	0	0	
Tumor encapsulation	Complete	31 (83.8%)	30 (78.9%)	1
	Incomplete	4 (10.8%)	4 (10.5%)	
	N/A	2 (5.4%)	4 (10.5%)	
Intrahepatic metastasis	Absent	13 (35.1%)	5 (13.2%)	0.056
	Present	24 (64.9%)	32 (84.2%)	
	N/A	0	1 (2.6%)	
HBsAg	Positive	29 (78.4%)	33 (86.8%)	0.375
	Negative	8 (21.6%)	5 (13.2%)	
	N/A	0	0	
AFP	< 400	18 (48.6%)	27 (71.1%)	0.061
	> = 400	19 (51.4%)	11 (28.9%)	
	N/A	0	0	

Note: N/A: Not available. *P*-values were calculated by chi-square test or Fisher's exact test. *P*-value of < 0.05 (two sided) was considered as significant and written in bold text.

### NTCP expression level is significantly higher in the HBV cccDNA positive tumor tissues

Since NTCP has been shown to be a functional cellular receptor responsible for HBV entry into hepatocytes [9], we then investigated whether the lower/undetectable NTCP expression in tumor tissues could affect the HBV cccDNA reservoirs in HCC. To do this, we firstly detected the HBV cccDNA levels in the tumor tissues and paired adjacent non-tumor tissues in a partially overlapping cohort of 76 HBV-HCC patients. Though the positive detection rate for HBV cccDNA showed no statistically significant difference between tumor tissues and paired adjacent non-tumor tissues (59.21% *vs*. 68.42%, *P* = 0.2374), the copy number of HBV cccDNA in tumor tissues was significantly lower than that in the paired adjacent non-tumor tissues (0.25 copy/cell *vs*. 2.00 copy/cell, *P* = 0.0243, Table 2). Next, we analyzed the relationship between HBV cccDNA and NTCP mRNA levels in 49 patients with both NTCP expression and HBV cccDNA copy number data. Based on the HBV cccDNA status in tumor tissues, these patients were sub-divided into two groups: 31 were classified as cccDNA positive, and the remaining 18, who had undetectable levels of HBV cccDNA were classified as cccDNA negative. Application of the Krustal-Wallis test revealed that NTCP expression levels were significantly higher in the cccDNA positive group than that in cccDNA negative group (1.15 ± 1.37 vs. 0.43 ± 0.56, *P* = 0.0366). Taken together, these results are consistent with our previously described results that HCC tissues express lower level of NTCPs, which may limit HBV infection and thus contain less cccDNA reservoir. On the other hand, the paired adjacent non-tumor tissues remained to be susceptible to HBV infection due to higher level of NTCPs, as such contained higher level of cccDNA reservoir.

**Table 2:** The copy number of cccDNA in 76 pairs of HCC tissues

	cccDNA positive case n (%)	Median of cccDNA copy (/cell)
**Tumor tissue**	45(59.21%)	0.25
**Non-tumor tissue**	52 (68.42%)	2.00
***P* value**	0.2374	**0.0243**

*P* values were calculated by Krustal-Wallis test. *P* value of < 0.05 (two sided) was considered as significant and written in bold text.

### Ectopic expression of NTCP suppressed proliferation and growth of HCC cell lines

**Figure 2:**
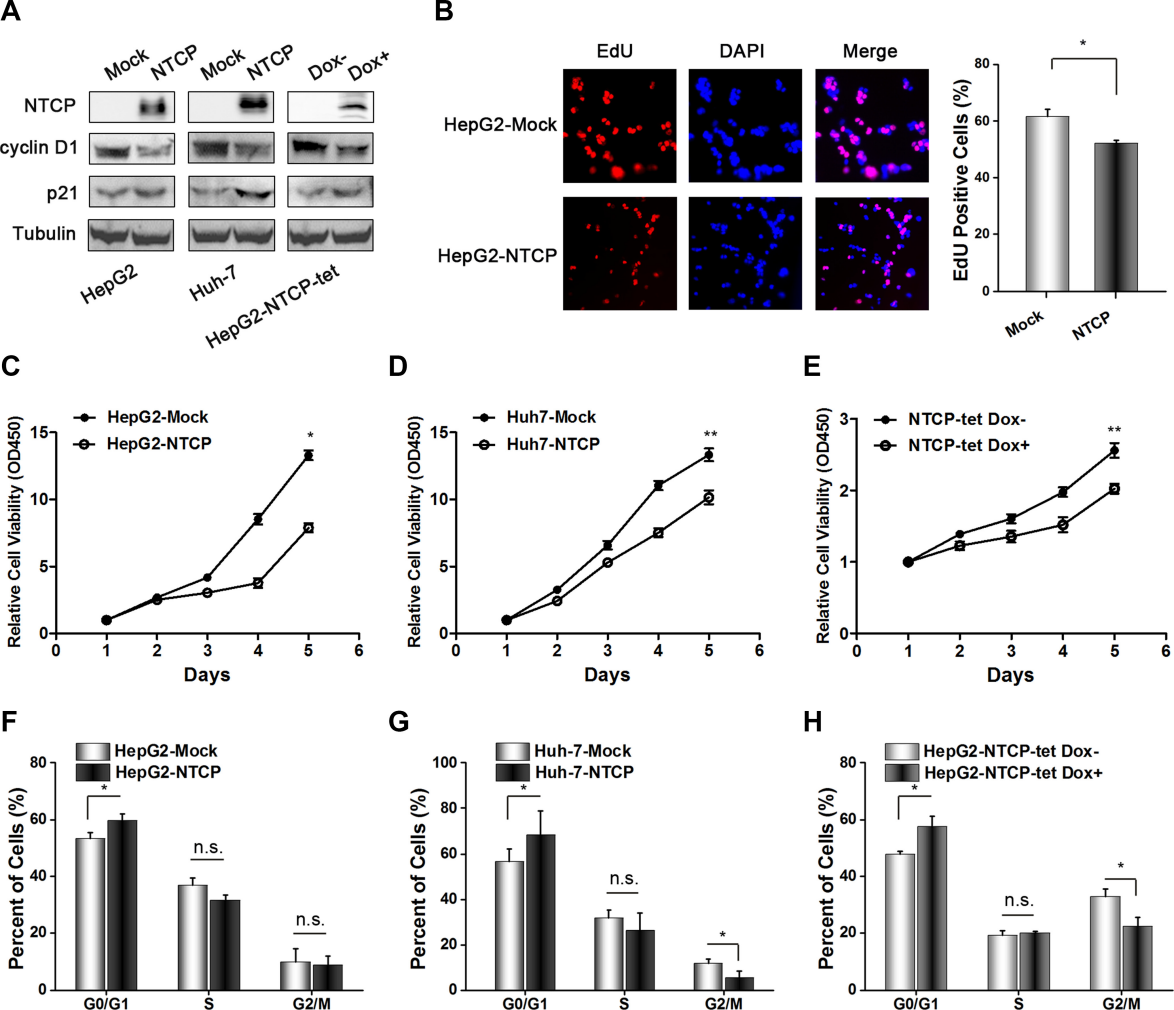
Ectopic NTCP expression suppresses proliferation in HCC derived cell lines (**A**) Detection of NTCP expression levels in HCC derived cell lines by western blotting, and the protein levels of cyclin D1 and p21 were also analyzed. Flag tag antibody and NTCP monoclonal antibody were used to detect NTCP expression. (**B–E**) Cell proliferation analysis in cells either stably expressing ectopic NTCP or expressing it following Dox mediated induction in NTCP-tet cells. In panel B, the EdU Cell Proliferation Assay was conducted in HepG2 cells stably expression NTCP. In panel C, D and E, CCK-8 assays were used to detect cell proliferation. Data are shown as the mean ± standard deviation of 6 repeats in three independent experiments. (**F–H**) Flow cytometry analysis of the cell cycle was conducted in the cells either stably expressing NTCP or expressing it following Dox mediated induction in NTCP-tet cells. All experiments were repeated three times.

Given the poor survival rate and larger size of tumor tissue mass in HCC patients with lower NTCP expression, it is reasonable to assume that NTCP may function as a tumor suppressor gene. To confirm this, we stably expressed NTCP in HCC derived cell lines HepG2 and Huh-7 and established a doxycycline (Dox)-induced Tet-On NTCP expression system. Then we analyzed the protein level of cyclin D1 and p21 after overexpression of NTCP, and found that the expression of cyclin D1 decreased while expression of p21 increased (Figure 2A). In addition, the subcellular of NTCP in ectopic expressed NTCP Huh-7 cells was also analyzed, and we found that NTCP is not only localized on the membrane but also spreaded throughout in the cytoplasm (Supplementary Figure S2). The EdU incorporation assay showed that NTCP could suppress the proliferation of HepG2 cells (Figure 2B). Consistently, ectopic expression of NTCP significantly inhibited the cell growth of both HepG2 and Huh-7 cells by using CCK-8 assays (Figure 2C and 2D). Furthermore, flow cytometry assays demonstrated that ectopic NTCP expression induced cell cycle arrest in G0/G1 phase in HepG2 and Huh-7 cells (Figure 2F and 2G). Additionally, doxycycline (Dox)-mediated regulated expression of NTCP in HepG2 NTCP-tet cells also led to a significant suppression of cell growth and obvious G0/G1 phase arrest (Figure 2E and 2H). Taken together, these results suggested that NTCP may function as a tumor suppressor gene and the down-regulation of NTCP may promote the development and progression of HCC.

### Cyclin D1 transcriptionally inhibited NTCP expression in HCC cell lines

**Figure 3:**
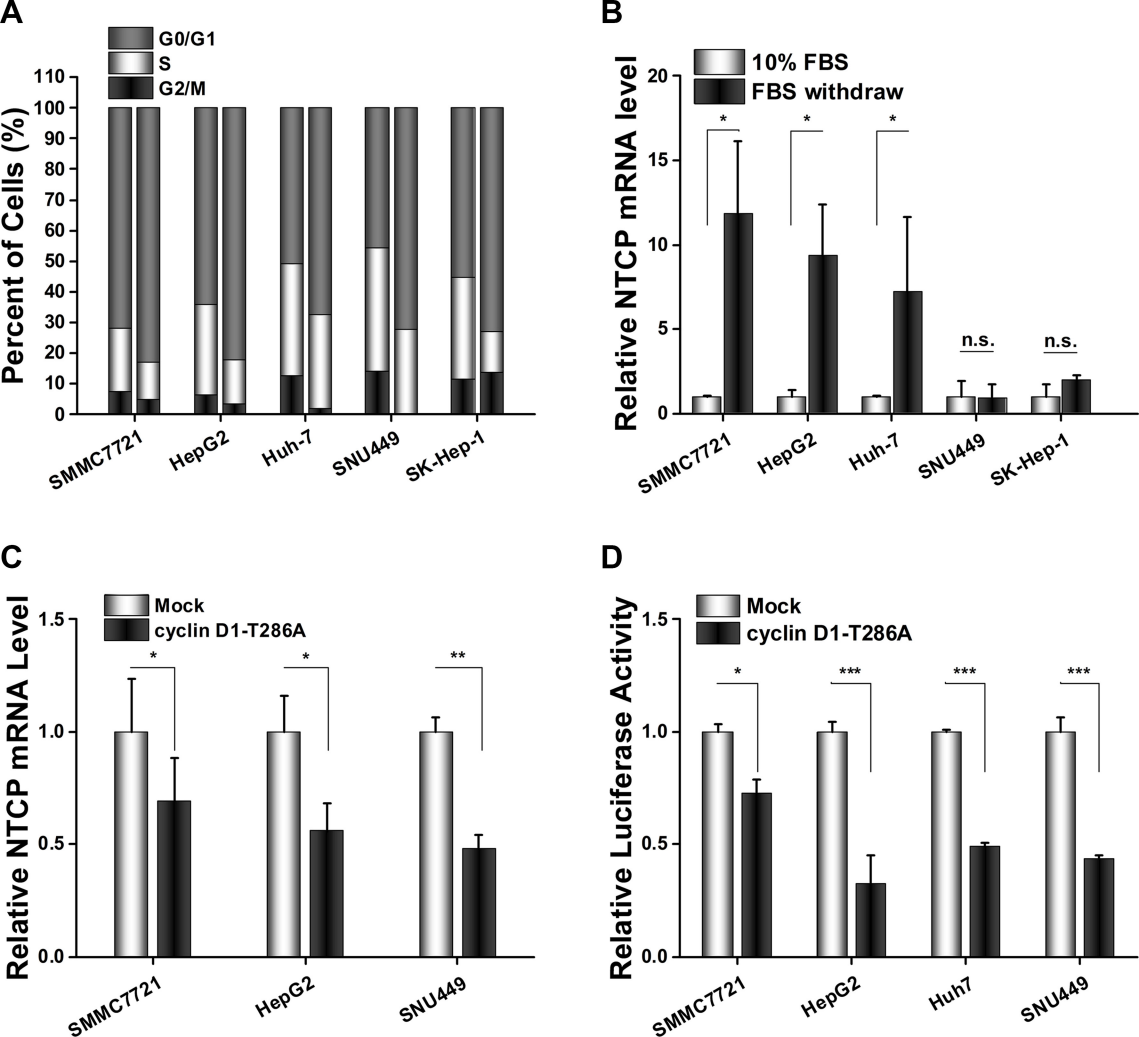
Cyclin D1 suppressed NTCP expression by inhibiting the transcriptional activity of NTCP promoter in HCC cell lines (**A**) Flow cytometry cell cycle assays carried out before and after serum starvation (left columns show normally proliferating controls and the right columns are of cells arrested in G0/G1 by serum starvation). (**B**) NTCP mRNA levels were detected by real-time RT-PCR in HCC cell lines treated as (A). After normalization against CTBP1, the values of relative NTCP mRNA level in cells cultured with 10% serum were designed as 1. (**C**) Real-time RT-PCR analysis of NTCP mRNA levels in HCC cells which transfected with either cyclin D1-T286A expression plasmid or an empty pFLEX vector. (**D**) Luciferase reporter assays were conducted to analyze the activity of NTCP promoter. Cells were co-transfected with NTCP promoter reporter constructs and cyclin D1-T286A expression plasmid or empty pFLEX plasmid. (*P < 0.05; **P < 0.01; ***P < 0.001; n.s.: not significant.).

To investigate the underlying mechanism of NTCP down-regulation in HCC tissues, four HCC cell lines and one adenocarcinoma originated cell line SK-Hep-1 were arrested in the G0/G1 phase by culturing in serum-free culture medium for 72 hours. Flow cytometry assays confirmed that most cells were arrested at the G0/G1 phase following this serum starvation (Figure 3A). Then, the levels of NTCP mRNA in these G0/G1 phase arrested cells and their normally proliferating counterparts were tested using real-time RT-PCR assays. As shown in Figure 2B, three of the HCC cell lines analyzed (HepG2, SMMC7721 and Huh-7) exhibited a 7 to 12-fold increase in NTCP mRNA levels in G0/G1 arrested cells (Figure 3B); whereas the NTCP mRNA levels remained unchanged by G0/G1 arrest in SNU449 and SK-Hep-1 cells. These data demonstrated that NTCP expression was inhibited in some proliferating HCC cells, which was consistent with the down-regulation of NTCP seen in HCC tumor tissues.

Cyclin D1 is a key player in cell cycle progression, it is critical for driving cells from G0/G1 phase to S phase. To detect whether cyclin D1 attributed to the NTCP down-regulation in proliferating cells, we transfected the constitutively active T286A mutant of cyclin D1 into these HCC cell lines and detected the levels of NTCP expression by real-time RT-PCR assays. The results showed that NTCP mRNA levels in HCC cell lines were significantly down-regulated following overexpression of the T286A mutant of cyclin D1 (Figure 3C). To examine the regulation of cyclin D1 on the transcriptional activity of NTCP promoter, a conserved active region of the NTCP promoter was cloned and its activity was analyzed by using Luciferase reporter assays following co-transfection with cyclin D1-T286A mutant. As expected, ectopic expression of the cyclin D1-T286A mutant caused a significant decrease of the NTCP promoter activity in all four HCC cell lines tested (Figure 3D). This result suggested that cyclin D1 may exert its inhibition role by affecting the activity of NTCP promoter in proliferating HCC cells.

## DISCUSSION

NTCP is both a key cellular transporter mediating the uptake of bile acids into hepatocytes and an essential receptor for HBV infection. Zollner G. et al. have reported that NTCP expression was decreased in HCC tumor tissues [13]. Consistent with this report, our study also demonstrated that the NTCP expression level in HCC tumor tissues was significantly lower than that in the adjacent non-tumor tissues. More importantly, we found that lower levels of NTCP expression in tumor tissues was associated with poor post-surgery survival rate in HCC patients and ectopic expression of NTCP in HCC cell lines could inhibit cell proliferation and growth. These observations strongly suggest that NTCP may function as a potential tumor suppressor gene.

Expression of NTCP is maintained at a steady level in well differentiated adult hepatocytes. To date, a series of transcript factors have been identified to regulate the expression of NTCP, including HNF1α, HNF4α, HNF3β-I, HNF3β-II, CEBP-α/β etc. [16–18]. However, the detailed mechanism(s) involved in NTCP down-regulation in HCC tumor tissues is still elusive. Miura et al. observed a dynamic changes of NTCP expression after the 90% partial hepatectomy in rat liver, revealed an association between NTCP transcription regulation and the proliferation status of hepatocytes [14]. This finding was supported by the fact that NTCP expression was down regulated in HCC tumor tissues in which unlimited proliferation potential is frequently observed. In this study, we showed that levels of NTCP mRNA were significantly increased when the proliferating HCC cell lines were forced into G0/G1 phase by serum starvation, providing direct evidence *in vitro* that NTCP expression is regulated by cell cycle progression.

Although the proliferation status of hepatocytes showed a close association with the NTCP expression, little was known about the potential mechanism(s) involved. This study is the first to demonstrate that NTCP expression is transcriptionally inhibited by cyclin D1. Cyclin D1 is the key regulator of G1 phase reentry and cell cycle progression. During G1 phase, cyclin D1 accumulates and interacts with either cyclin-dependent kinase 4 or 6 (CDK4 or CDK6) in response to mitogenic growth factors. Active cyclin D1/CDK4 complexes could translocate to the nucleus and phosphorylate the retinoblastoma protein (Rb) and its related family members, thereby triggering E2F-dependent transcription of genes required for S phase entry [19–22]. To investigate the underlying mechanism of cyclin D1 in regulating the transcriptional activity of NTCP, we analyzed the highly conserved 5′-UTR promoter sequence of NTCP gene and identified a potential E2F binding site in the 5′-UTR of NTCP promoter (Supplementary Figure S3). We demonstrated that the constitutively active T286A mutant of cyclin D1 could significantly inhibit the transcriptional activity of the NTCP promoter. However, mutation in E2F recognition site of NTCP promoter could not reverse the inhibition of cyclin D1-T286A on NTCP transcriptional activity (data not shown). This result suggested that cyclin D1 may not exert its inhibiting role by directly interacting with the potential E2F binding site in the NTCP promoter. Further study should explore the precise mechanisms which cyclin D1 inhibits NTCP promoter activity. In addition, a series of studies had revealed that cyclin D1 was overexpressed in HCC or accumulated in nuclear of hepatocytes [23]. Thus, it was reasonable to postulate that the NTCP down-regulation in HCC tumor tissues might be due at least in part to cyclin D1 overexpression.

The presence of NTCP has been shown to be essential for HBV infection [9]. In our study, the copy number of HBV cccDNA in HCC tumor tissues with lower NTCP expression was found to be lower than that in the adjacent non-tumor tissues with higher NTCP expression. This data supports the role of NTCP in the HBV entry, even importantly, lower or depleted NTCP expression may endow newly produced hepatocytes with resistance to HBV infection [24] and hence suppression of NTCP expression might be a strategy to protect newly formed hepatocytes from being infected by HBV. A decrease in NTCP-mediated HBV entry into constitutively proliferating hepatocytes in HCC may result in dilution of the cccDNA pool, which could result in a decrease of cccDNA reservoirs in malignant transformed hepatocytes. These considerations are a timely reminder that to achieve the eventual clearance of cccDNA from hepatocytes during the antiviral therapy, it will be important to focus both on inhibiting HBV viral replication and protecting newly divided hepatocytes from viral infection.

In conclusion, this is the first study to report that the transcriptional inhibition of NTCP expression during cell cycle progression was mediated by cyclin D1. NTCP may function as a potential tumor suppressor gene in HCC and the down-regulated NTCP expression was associated with poor prognosis and lower HBV cccDNA copy numbers in HCC patients. Therefore, these results will help us to further understand the mechanisms of the poor cccDNA reservoirs seen in HBV-mediated HCC tumor tissues, and provide evidence that NTCP expression levels might serve as a novel prognostic biomarker for post-surgery survival rate of HCC patients.

## MATERIALS AND METHODS

### HCC cell lines and cell transfection

Human hepatocellular carcinoma cell lines HepG2, Huh-7, SNU449 and the human liver adenocarcinoma cell line SK-Hep-1 were purchased from the American Type Culture Collection (Manassas, VA, USA). Human HCC cell line SMMC7721 was purchased from Cell Resources Center of Peking Union Medical College (Beijing, P.R. China). Cells were transfected with plasmids using Lipofectamine 2000/3000 (Invitrogen, Carlsbad, CA, USA) according to the manufacturer's instructions.

### Patient specimens

Pairs of primary human HCC tumor and adjacent non-tumor tissues were obtained from patients who underwent surgical resection in the Affiliated Oncology Hospital of Zhengzhou University between October 2012 and October 2014. Informed consent was obtained from each patient prior to participation, and this study was approved by the Ethics Committees of Peking University Health Science Center.

### Real-time reverse transcription (RT)-PCR

Real-time RT-PCR was performed as described previously [25]. C-terminal binding protein 1 (CTBP1) was used as the reference gene to determine gene(s) expression in HCC [26]. The primers used for real-time RT-PCR are shown as follows: NTCP-forward: 5′ - TGAC CACCTGCTCCACCTTC -3′ and NTCP-reverse: 5′– GAA TGAGAACCAGGACCAGTGAT -3′; CTBP1-forward: 5′ - TTCACCGTCAAGCAGATGAGAC-3′and CTBP1-reverse: 5′ - CTGGCTAAAGCTGAAGGGTTCC-3′.

### HBV cccDNA detection

HBV cccDNA from liver tissues were detected as described [27]. In brief, HBV cccDNA was detected in the way of combining PSAD digestion, rolling circle amplification and Taqman Probe RT-PCR. Primers, and the method can be described as following. The first step is to section formalin fixed paraffin-embedded (FFPE) liver tissue to extract DNA using QIAamp FFPE DNA Mini Kit (QIAGEN, Hilden, Germany) according to the manufacturer instructions. Secondly, PSAD (Epicentre, Madison, WI, USA) was used to digest HBV DNA. Afterwards, Rolling Circle Amplification (RCA) was launched to selectively amplify circle DNA. Next, the RCA products was used as templates to amplify HBV covalently closed circular DNA (cccDNA), and then quantified with TaqMan real-time PCR by a pair of cccDNA-selective primer and a probe that targets the gap region between the two direct repeat regions (DR1 and DR2) of the HBV genome. Finally, to quantitate hepatocytes numbers, a set of primers and a probe for reference control DNA segment of human beta-actin were used in the real-time PCR. Cell numbers were calculated based on an estimation of 6.667 pg/hg DNA per cell.

### Luciferase reporter assay

The conserved 5′-UTR of human NTCP promoter was amplified by PCR using human genomic DNA as template and inserted into pGL3-Basic plasmid. The primers used to amplify NTCP promoter are as follows, sense: 5′-CCT CGAGGTGACAAGGGAGGAGTACAAGTAGCACCC AG-3′; anti-sense: 5′- CAAGCTTGCTCCATCCTCCTG TGAGGCAGTGGAAGACCACTC -3′. Dual-luciferase reporter assays were carried out as described [25]. In brief, cells were seeded in 12-well plates and co-transfected with 0.3 μg of firefly luciferase reporter plasmids together with 1 μg pFLEX-cyclinD1-T286A expression plasmids and 10 ng of actin-Renila expression plasmids using Lipofectamine 2000/3000 in each well. The cell's luciferase activity was quantified 36 hours after transfection using a dual luciferase reporter kit (Promega, Madison, WI, USA), following the manufacturer protocol.

### Generation of mAb P17-39 against human NTCP

The NTCP mAb, P17-39, was generated in house. Briefly, 293T cells was transiently transfected with a human NTCP expressing plasmid and confirmed high expression of NTCP by FACS using a mAb against the tag fused to the C-terminal of NTCP. The 293T-NTCP cells were then used to immunize mice. Mouse mAbs was generated by standard hybridoma technology. By using opera^®^ high content screening system (PerkinElmer), mAbs specifically recognizing NTCP were screened out based on the specific binding to NTCP expressing cells but not the control cells lacking NTCP expression. P17- 39 was one of the mAbs against NTCP screened out. It was further characterized for specific binding with NTCP by Western blot, FACS and immunofluorescent staining (Supplementary Figure S1).

### Western blot analysis and IHC of liver tissues from HCC patients

Western blot analysis was performed similarly as previously described [25]. Briefly, the lysed cell supernatant was run a SDS-PAGE, and blotted with P17- 39, cyclin D1 (MBL), p21 (MBL) and α-Tubulin (MBL) at 0.5∼1 μg/mL, then detected by FITC labeled Goat Anti-mouse secondary antibody (Life). IHC staining was performed as following. In brief, 5 μm thick liver tissues microarray sections were deparaffinized and rehydrated. Antigens retrieval were performed by antigen retrieval solution (BioGenex, San Ramon, CA) with a high pressure cooker for 30 min. Slides were blocked by Goat serum, then blotted with P17-39 at 5 μg/mL. The sections were then incubated with the secondary antibody followed by avidin-biotin-peroxidase complex. The chromogen substrate, NovaRed, was added and finally counter-stained with hematoxylin.

### Cell cycle assay

For cell cycle analysis, cells were harvested by trypsinization and fixed with 70% ethanol at 4°C overnight. The fixed cells were re-suspended in propidium iodine (PI) solution containing 50 μg/mL RNase A (Sigma, USA), and incubated at 37°C for 30 min in the dark. The fluorescence of the PI labeled cells was then measured using a flow cytometer (FACS Calibur, BD Biosciences, San Jose, CA, USA).

### Cell Counting Kit-8 (CCK8) assays

Cells were seeded in 96-well plates at a density of 1.5 × 10^4^ per well. 24 hours later, CCK-8 (Dojindo Laboratories, Rockville, MA, USA) reagent (10 μl/well) was added and incubated for 1 hr. Following vortexing for 5 mins, the absorbance value of each well was measured at 450 nm. Each sample was tested in six repeats in three separate experiments.

### Edu incorporation assay

Cells were seeded into 96-well plates and incubated under standard conditions in complete media. 24 hours later, cell proliferation was detected using the EdU Cell Proliferation Assay Kit (Ribobio, Guangzhou, China). Images of cells were captured with Leica fluorescent microscope equipped with camera and analyzed by Photoshop software. In each individual experiment, at least three fields were randomly selected from captured images, and the average nuclear fluorescent intensity was calculated.

### Statistical analysis

The Student's *t*-test was used to compare two groups of continuous variables. Kaplan-Meier curves were used to assess overall survival rate. All estimates were accompanied by a 95 % confidence interval, differences were considered statistically significant at *P* < 0.05.

## SUPPLEMENTARY MATERIALS FIGURES AND TABLES


