# Supplementary material for: Down-regulation of NTCP expression by cyclin D1 in hepatitis B virus-related hepatocellular carcinoma has clinical significance
Source: Oncotarget. 2016 Jun 23;8(34):56041–50. doi: 10.18632/oncotarget.10241 (PMC5593543; doi:10.18632/oncotarget.10241)
Supplement: Supplementary file 1 [file oncotarget-08-56041-s001.pdf]

Down-regulation of NTCP expression by cyclin D1 in hepatitis B virus-related hepatocellular carcinoma has clinical significance

Supplementary Materials

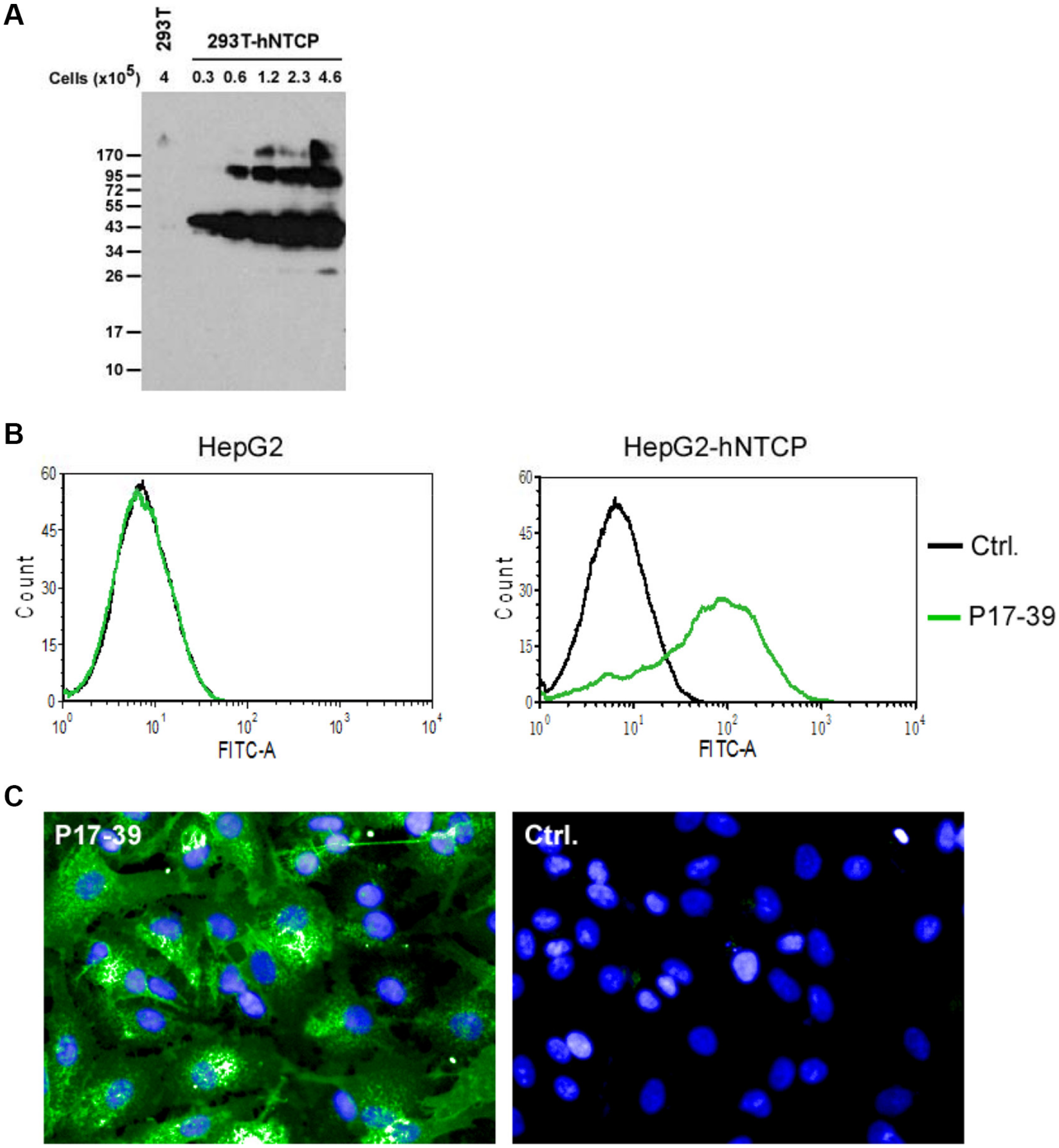

Supplementary Figure S1: Characterization of mouse mAb against human NTCP (hNTCP), P17-39. (**A**) Western blot of NTCP expression on 293T cells by P17-39 mAb. 293T-hNTCP cells were 293T cells transiently transfected with NTCP expressing plasmid. 293T cells transfected with empty vector were used as control. Cells were harvested 48 hours after transfection and lysed in RIPA buffer on ice for 2 hrs. The lysed cell supernatant (corresponding to the initial cell number as indicated) was then run a SDS-PAGE, and blotted with P17-39 at 0.5~1 µg/mL, detected by HRP-anti mouse secondary antibody (Sigma). (**B**) FACS analysis. The human NTCP expressing stable cell line HepG2-hNTCP and control HepG2 cells were stained with 5 µg/mL of P17-39 mAb, then detected by FITC anti-mouse secondary antibody (Sigma). (**C**) Immunofluorescent staining. The human NTCP expressing stable cell line HepG2-hNTCP and control HepG2 cells were first fixed and permeabilized, then stained with 5 µg/mL P17-39, followed by FITC anti-mouse secondary antibody (Sigma) and DAPI to stain NTCP and cell nuclei.

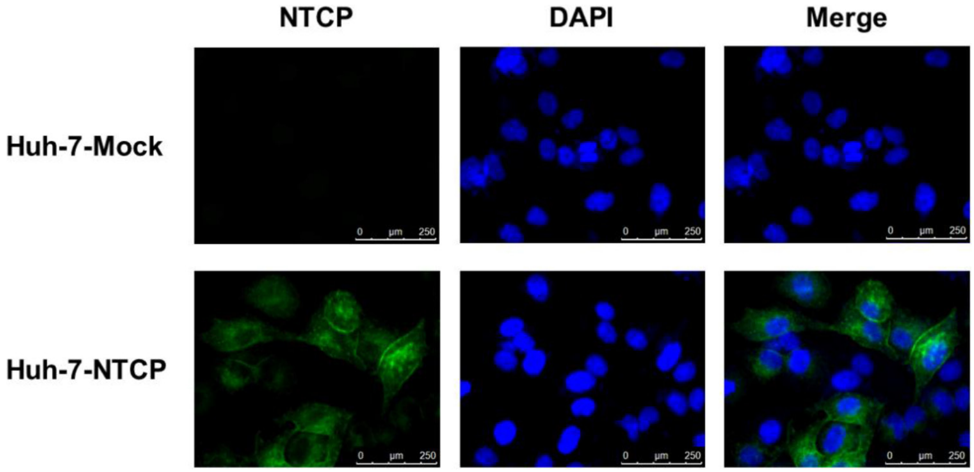

Supplementary Figure S2: Subcellular localization of NTCP in NTCP stably expressed Huh-7 cells. Immunofluorescence was conducted in the ectopic expressed NTCP Huh-7 cells and control Huh-7 cells, these cells were first fixed and permeabilized, then stained with 5 μg/mL P17-39, followed by FITC anti-mouse secondary antibody (Sigma) and DAPI to stain NTCP and cell nuclei.

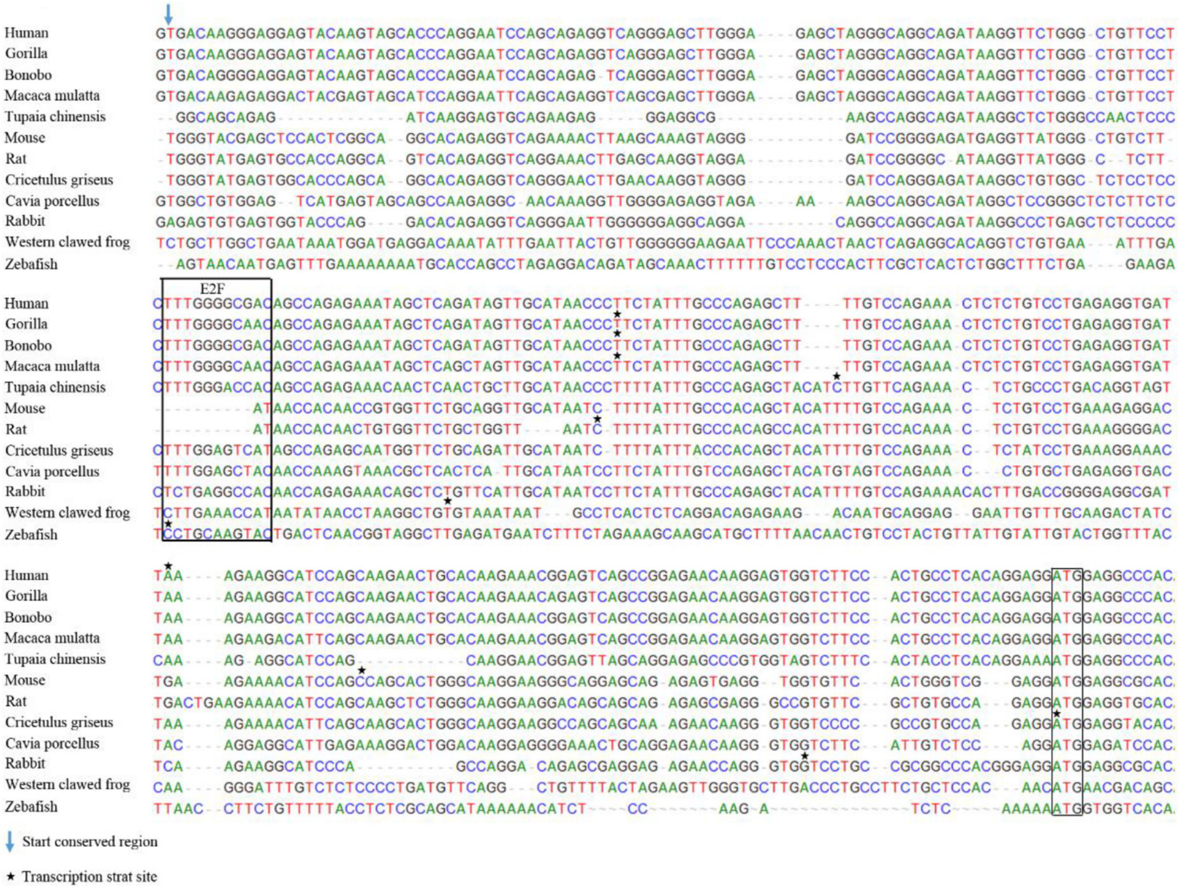

Supplementary Figure S3: Analysis of the conserved 5′-UTR of the NTCP gene from different species, including human, gorilla, bonobo, macaca mulatta, tupaia chinensis, mouse, rat, cricetulus griseus, cavia porcellus, rabbit, western clawed frog and zebrafish. Using computer analysis of previous reports, a highly conserved region of the NTCP promoter was found. Putative E2F recognition sites and translation start sites are outlined. *Start site of the published NTCP cDNA sequence (the transcription start sites of cavia porcellus and zebrafish are not included in this region).
